# The Effect of the Combination of Probiotics and Heavy Metals From Various Aspects in Humans: A Systematic Review of Clinical Trial Studies

**DOI:** 10.1002/hsr2.70521

**Published:** 2025-03-18

**Authors:** Atieh Darbandi, Tahereh Navidifar, Maryam Koupaei, Roghayeh Afifirad, Reyhaneh Amin Nezhad, Amir Emamie, Malihe Talebi, Maryam Kakanj

**Affiliations:** ^1^ Molecular Microbiology Research Center Shahed University Theran Iran; ^2^ Department of Basic Sciences Shoushtar Faculty of Medical Sciences Shoushtar Iran; ^3^ Department of Microbiology, School of Medicine Tehran University of Medical Sciences Tehran Iran; ^4^ Department of Microbiology, Faculty of Biological Sciences Alzahra University Tehran Iran; ^5^ Department of Pathobiology, School of Public Health Tehran University of Medical Sciences Tehran Iran; ^6^ Microbial Biotechnology Research Centre Iran University of Medical Sciences Tehran Iran; ^7^ Department of Microbiology, School of Medicine Iran University of Medical Sciences Tehran Iran; ^8^ Food and Drug Laboratory Research Center Food and Drug Administration, MOH&ME Tehran Iran

**Keywords:** detoxification, diarrhea, heavy metals, iron deficiency, probiotics

## Abstract

**Background and Aims:**

Probiotics usually have beneficial effects on the absorption of trace elements and detoxification of toxic metals in human. Hence, the aim of the systematic review was to evaluate various aspects of the effect of the combination of probiotics and heavy metals in human clinical trial studies.

**Methods:**

Nine databases were searched for clinical trials up to June 2024 investigating probiotics for heavy metal exposure in humans. Two reviewers independently screened records and extracted data on study characteristics, interventions, outcomes, and results. Risk of bias was assessed.

**Results:**

The analysis included 31 clinical trials with a total of 4,611 participants, focusing on the effects of probiotics, prebiotics, and synbiotics. Among the trials, 23 investigated probiotics, five looked at prebiotics, and three explored synbiotics, with probiotic doses ranging from 10^7^ to 2.5 × 10^10^ CFU/day. Results indicated that probiotics combined with zinc significantly reduced the time to resolution of vomiting and diarrhea compared to zinc alone, improved the treatment efficacy of antibiotic‐associated diarrhea linked to pneumonia, and shortened hospital stays relative to probiotics alone. Probiotics did not show significant effects on blood parameters compared to placebo; however, prebiotic galactooligosaccharides enhanced iron absorption in women and children. The addition of probiotics to bismuth quadruple therapy did not improve *Helicobacter pylori* eradication rates but reduced side effects like diarrhea and vomiting. One trial reported a decrease in toxic metal levels in pregnant women due to probiotics, but no similar effects were observed in children.

**Conclusions:**

Probiotics are one of the new methods employed to improve or eliminate the adverse effects of heavy metals in the body. Although many studies have investigated the effects of probiotics on heavy metals, there is still a need for more in‐depth and extensive studies.

## Summary

- Toxic heavy metals can harm health, probiotics detoxify.
- Probiotics + zinc shorten diarrhea, reduce side effects of H. pylori therapy.
- Probiotics may help reduce heavy metal toxicity; more research needed for confirmation.

## 1 Introduction

Probiotics are live microorganisms that, when administered in appropriate doses, provide health benefits to the host. Probiotics have become popular with the public because of their potential for disease prevention and treatment, cost‐effectiveness, and accessibility [1]. Probiotics work through a variety of mechanisms, although the exact mechanism remains unclear. These include production of bacteriocin and short‐chain fatty acids, reduction of intestinal pH, nutrient competition, improvement of mucosal barrier function, and immunomodulation [2].

Probiotics such as *Lactobacillus acidophilus*, *Saccharomyces cerevisiae, L. fermentum* and *L. rhamnosus* usually have beneficial effects on the absorption of trace elements such as iron, copper and zinc [3]. Some probiotics such as *L. plantarum, Bacillus cereus, L. reuteri*, and *L. brevis* are also able to detoxify toxic metals such as lead, cadmium, mercury, and arsenic in humans [4].

There is substantial evidence to support the use of probiotics in the treatment of acute diarrhea [5]. The World Health Organization has also recommended the use of oral zinc to treat acute gastroenteritis [6]. Zinc as a micronutrient is important in reducing the duration and severity of diarrhea. Therefore, some studies have evaluated the effect of combining zinc and probiotics in the treatment of diarrheal diseases [7, 8].

Probiotic can be a clinical tool to optimize dietary iron bioavailability to improve iron status without the gastrointestinal burden of additional supplemental iron. Vonderheid et al. showed in a meta‐analysis that probiotic *L. plantarum* was effective in significantly increasing nonheme dietary iron absorption [9]. *L. plantarum* is thought to positively affect dietary nonheme iron absorption through several mechanisms, including (1) the production of p‐hydroxyphenyllactic acid, a microbial by‐product, that can enhance the conversion of ferric iron to the more readily available ferrous form [10]; (2) the enhanced iron uptake into enterocytes through increased mucin production [11]; and (3) immunomodulation through the suppression of hepcidin production, resulting in the increased iron bioavailability [12].

In recent years, there has been growing concern about the increasing prevalence of *H. pylori* resistance. There is some evidence on the anti‐*H. pylori* properties of certain probiotic bacteria during in vitro experiments [13]. On the other hand, Bismuth is well recognized for its antibacterial properties, effectively preventing bacterial colonization of gastric epithelial cells, with no documented resistance to *H. pylori* [14]. Therefore, it is hypothesized that the *Lactobacillus* spp. such as *L. reuteri* and *L. acidophilus* combination with bismuth containing quadruple therapy regimen may have a synergism effect effective in the eradication of *H. pylori* [15].

Currently, probiotics are recognized as a potentially beneficial candidate for reducing heavy metal toxicity. Probiotic strains of *Lactobacillus* have the capability to sequester heavy metals, thus serving as valuable agents in heavy metal detoxification processes [16]. In addition, these particular strains have the ability to promote the elimination of heavy metals through fecal excretion, thereby counteracting the changes in gut microbiota induced by heavy metal exposure [4]. The detoxification mechanism of heavy metals by probiotics involves the binding of metallic ions to the bacterial cell wall, leading to their accumulation within the bacteria [17]. Moreover, probiotic bacteria have the capability to convert more toxic forms of heavy metals into less toxic ones. For instance, lactobacillus bacteria can convert methylated mercury into inorganic mercury (Hg2 + ), and subsequently into Hg0, a form that exhibits low absorption in the gastrointestinal tract [18].

According to our knowledge, there is no systematic review to evaluate various aspects of the effect of the combination of probiotics and heavy metals in human. Therefore, the aim of this article was to review these various aspects when probiotics were combined with heavy metals in human clinical trials.

## 2 Materials and Methods

### 2.1 Literature Search Strategy

This study searched nine international databases (Medline, Science Direct, Scopus, Web of Science, Embase, Cochrane Library, ProQuest, Open Grey, and Google Scholar) for eligible articles published in English up to June 2024. Also, our search alert in PubMed was on for articles published up to June 2024. The search strategy was based on a combination of the following key terms: “heavy metal” AND “probiotics” OR “prebiotics” OR “synbiotics”. Two reviewers independently screened the title and abstract of each study, and additional articles were identified by screening the reference lists of selected articles and relevant reviews. EndNote removed duplicates. Finally, the full text of articles deemed to be potentially eligible was retrieved for further detailed evaluation.

### 2.2 Inclusion and Exclusion Criteria

**Figure 1:**
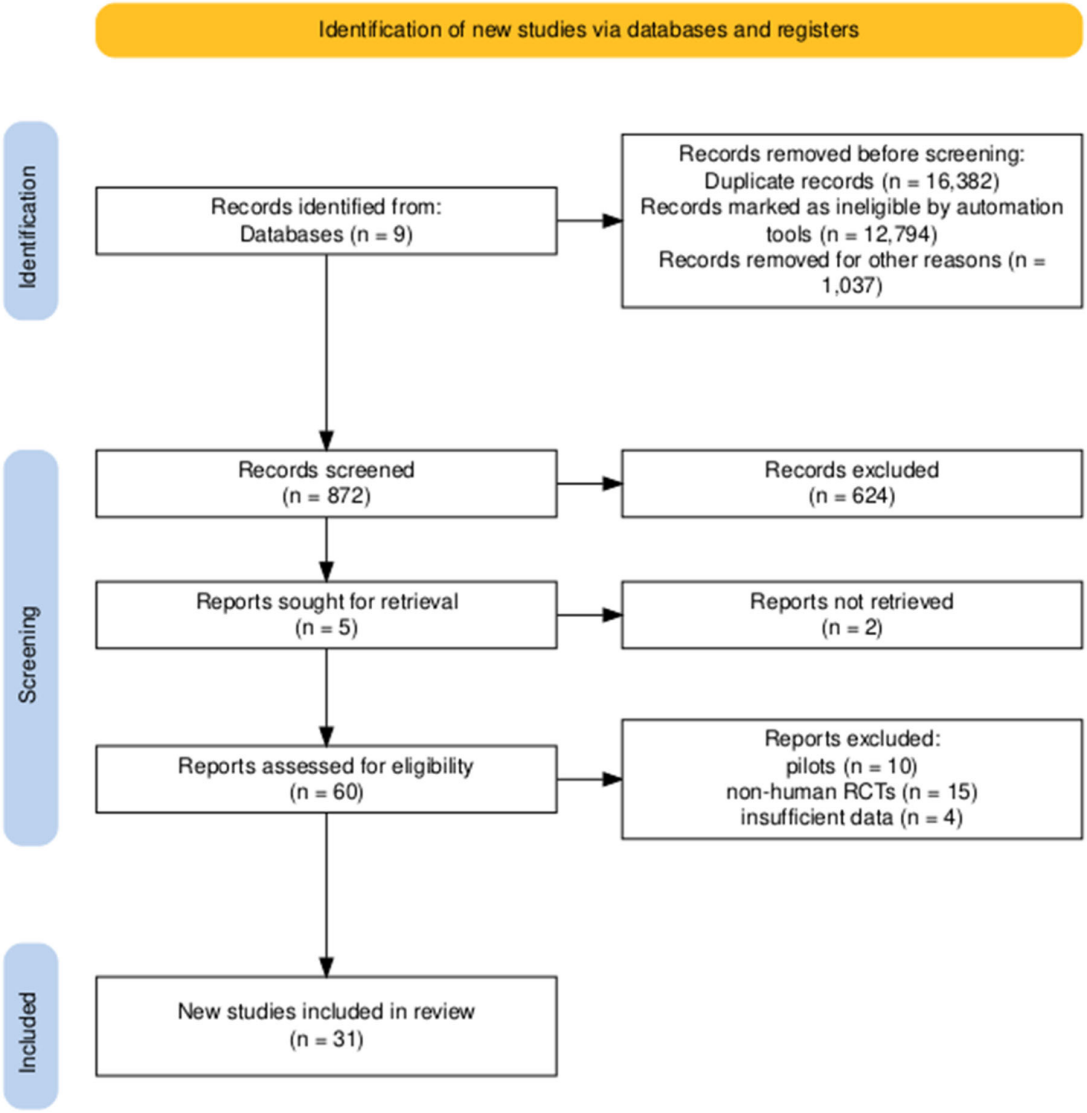
Flowchart of the article's selection procedure for this systematic review based on PRISMA.

This systematic review was conducted according to the Preferred Reporting Items for Systematic Reviews and Meta‐Analyses (PRISMA) guidelines (2020). Studies had to meet the following criteria to be included in this study: (1) well‐described, randomized, controlled trials (RCTs) with high quality and defined outcomes, (2) which were performed on human participants who were exposed to or consumed heavy metals and (3) received supplementary probiotic, prebiotic, or synbiotic interventions. Non‐English articles, nonhuman trials, preliminary studies without full text, duplicate reports, narrative reviews, comments, opinion pieces, methodological reports, or conference abstracts were excluded from the study. Figure 1 depicts the flow diagram of the literature search and article selection.

### 2.3 Article Selection, Quality Assessment, and Data Extraction

The initial phase of article selection was based on analysing titles and abstracts to identify articles relevant to the scope of this research. Reference lists of all selected publications were investigated to find any ignored articles, and those publications cited in more than one database were included once. Then the full text of selected articles was then retrieved, and studies were subjected to full‐text evaluation based on the eligibility criteria.

Two reviewers extracted, screened, and analyzed data independently, and consensus resolved discrepancies. A third researcher checked the results to ensure that all the eligible articles were evaluated. The information extracted from each included article was as follows: author's last name, country, sample size (intervention/control), mean age, mean weight, study design, participants' characteristics, intervention type (probiotics/synbiotics), probiotics species, intervention dose, intervention period, duration of follow‐up, and outcome.

## 3 Results

A total of 26,742 articles were retrieved by searching nine electronic databases. The summary of the article selection procedure is illustrated in the PRISMA flowchart (Figure 1). Some articles were excluded in the second screening phase based on their title and abstract evaluation. After excluding duplicates, filters, and articles not meeting the inclusion criteria, 452 full‐text articles remained, which were read carefully for further assessment, of which 430 articles were excluded as irrelevant. Overall, 31 articles with 4,611 participants (the age range of 1 month to 65 years) describing the effects of probiotics on heavy metal consumption were selected and included in this study for further analysis. The overall results of the 31 studies reviewed in this research are summarized in Table 1. Among the 31 clinical trials reviewed, 23, 5, and 3 examined the effects of probiotic, prebiotic, and synbiotic products, respectively.

**Table 1:** The outcomes of different clinical trials assessing the heavy metal tolerance and bioremediation through the use of pro/prebiotics.

First author and year	Country	Type of metal	No. of participants (mean age ± SD)	Study design	Participants characteristics	Probiotic, prebiotics	Dose	Intervention	Control used and duration of therapy	Outcomes
Shamir et al. (2005) [19]	Israel	Zinc	65 (9.42 ± 1.98 M)	RDBPCT	Infants with acute diarrhea	*S. thermophilus*	6 × 10^9^ CFU	Probiotics and prebiotics	Cereals (placebo)/600 mL/12 wks	Probiotics and zinc reduced the severity and duration of diarrhea
						*B. lactis*				
						*L. acidophilus*				
						FOS	0.3 gr			
Dalgic et al. (2011) [20]	Turkey	Zinc	480 (13.71 ± 6.210 M)	RSBCT	Patients with rotavirus diarrhea	*S. boulardii*	250 mg	Probiotic, o.d/< 5 d	ORS/o. D/ < 5 d	The use of zinc plus *S. boulardii* was beneficial for children and reduced the severity of diarrhea
Passariello et al. (2011) [21]	Italy	Zinc	119 (18.58 M)	RSBCT	Patients with acute diarrhea	FOS	0.35 g/l	1 sachet [super ORS (zinc + prebiotics)]/3‐4 h/72 h	1 sachet (standard ORS)/3‐4 h/72 h	The addition of zinc and prebiotics to ORS limits diarrhea duration in children
						XOS	0.35 g/l			
Agustina et al. (2013) [22]	Indonesia	Iron, zinc	494 (59.4 ± 14.3)	RDBPCT	Children	*L. beuteri* DSM 17938	5 × 10⁸ CFU	Low‐lactose milk/180 mL/b.i.d/24 wks	Placebo (vegetable oil)/b.i.d/24 wks	Probiotics and especially synbiotics decrease the concentration of triglyceride in pre‐diabetic adults
						*L. casei* CRL 431				
Shavakhi et al. (2013) [23]	Iran	Bismuth	170 (42.3 ± 13.3 yrs)	RTBPCT	Patients with *H. pylori* infection	*L. casei*	1 × 10^8^ CFU	1 capsule/b.i.d/2 wks	1 capsule/b.i.d/2 wks	Multi‐strain probiotic supplementation has no beneficial effects on treating *H. pylori* infection.
						*L. rhamnosus*			Placebo (magnesium stearate, hydroxypropyl methyl cellulose)	
						*L. acidophilus*				
						*L. bulgaricus*				
						*B*. *breve*				
						*B. longum*				
						*S. thermophilus*				
						FOS				
Bisanz et al. (2014) [24]	Canada	Mercury Lead Arsenic Cadmium	PW: 60 (23.5 ± 3.6 yrs)	ROLPS	Populations with high toxic metal exposures	*L. rhamnosus* GR‐1	1 × 10^10^ CFU	PW: 250 g yogurt/6 days a week/14 wks	PW: 4.3 g of Moringa/6 d/14 wks	Consumption of probiotics had a protective effect against further increases in mercury and arsenic blood levels in pregnant women, but this trend was not observed in children
			SAC: 44 (8.4 ± 1.2 yrs)							
								SAC: 250 g yogurt/6 days a week/25 days 3‐4 wks	SAC: ultra‐heat‐treated milk/25 days	
Randazzo et al. (2014) [25]	Italy	Nickel	22 (18 ‐65 yrs)	RDBPCT	Patients with an allergy to nickel	*L. reuteri* DSM 17938	1 × 10^8^ CFU	1 tablet/o.d/2 wks	1 placebo/o.d/2 wks	*L. reuteri* could be a helpful supplementation during a low‐Ni diet for patients with systemic nickel allergy syndrome
Srinarong et al. (2014) [26]	Thailand	Bismuth	100 (50.5 yrs)	PSCS	Patients with *H. pylori* infection	*B. lactis*	≥ 10^9^ CFU	Probiotic yogurt	Placebo (yogurt without probiotic)/1‐2 wks	Standard triple therapy plus bismuth and probiotic could provide an excellent cure for *H pylori* infection
						*L. acidophilus*		≥ 109 CFU/1‐2wks		
						*L. paracasei*				
Sheele et al. (2015) [27]	USA	Zinc	100 (39 ± 15 yrs)	RSBPCT	Cholera	*S. cerevisiae* var. *Boulardii*	Not reported	1 capsule/6 h/84 h	2 placebo capsules/6 h/84 h	Probiotic plus bismuth had no significant effect on reducing the duration and severity of cholera
		Bismuth								
Yazar et al. (2016) [28]	Turkey	Zinc	165 (36.4 ± 32.7 M)	SCRP	Acute diarrhea	(Fructose, GOS, polydextrose)	2.5 × 10^9^ CFU	1 sachet/d/+ prebiotic (1996.57 mg)/5 d	ORS/5 d	Zinc or synbiotic supplementation reduced the duration of diarrhea, and both could be used in children with acute diarrhea
						*L. casei*	4.5 × 10^9^ CFU			
						*L. rhamnosus*				
						*L. plantarum*				
						*B. lactis*				
Lazarus et al. (2017) [29]	India	Zinc	620 (5.1 ± 0.3 wks)	RDBPCT	Population receiving rotavirus vaccine	*L. rhamnosus* GG	1 × 10^10^ CFU	1 capsule/d/7wks	Placebo/1capsule/d/7 wks	A modest effect of combined supplements (probiotic + zinc)
Weinborn et al. (2017) [30]	USA	Iron	24 (37 ± 4 yrs)	RCT	Iron‐depleted women	Inulin, polydextrose,		200 mL bottle yogurt + prebiotic (2grams)/d/12 ds	200 mL bottle yogurt (without prebiotics)/d/12 ds	Prebiotics mix increases heme Fe bioavailability and does not affect nonheme iron bioavailability.
						Arabic gum and guar gum				
Xiang et al. (2018) [31]	China	Zinc	50	RCT	Antibiotic‐associated diarrhea secondary to childhood pneumonia	Group 1 Bifco containing *Nifidobacteria*, *L. acidophilus* and *E. faecalis* in combination Zinc, group 2: Bifco only	30 × 10^6^ CFU	One capsule	Not reported	Zinc combined with Bifico had significantly higher overall
							Zinc 20 mg daily for children > 6 mo and 10 mg for children < 6 mo	Three times per day for 14 days		Efficiency than Bifico alone for treatment of AAD secondary to pneumonia
Ferus et al. (2018)[32]	Poland	Iron	34 (10) yrs	RDBPCT	Iron deficiency anemia in patients with celiac disease	Inulin	Not reported	Prebiotic 10 g/d/12wks	Placebo (Maltodextrin) 7 g/d/12 wks	Oligofructose‐enriched inulin (Synergy 1) was a safe and well‐tolerated prebiotic in children and adolescents with CD in association with a GFD
Maragkoudaki et al. (2018)[33]	Greece	Zinc	51 (1.7 ± 0.7 yrs) 6–36 m	RDBPCT	Infants with acute diarrhea	*L. reuteri* DSM 17938	1 × 10^9^ CFU	1 sachet/d/4wks	1 sachet/d/4wks	ORS enriched with *L. reuteri* DSM 17938 and zinc was well tolerated with no adverse effects
Jeroense et al. (2019)[34]	Switzerland	Iron	34 (24.3 ± 3.2 yrs)	PSCCS	Iron‐depleted women	GOS	Not reported	Iron supplement with GOS (15 g/d) powder/4 wks	Iron supplement without single doses of GOS/4 wks	GOS, as a safe and widely used prebiotic consumed acutely with an iron dose, increases iron absorption from fefum
Poonyam et al. (2019) [35]	Thailand	Bismuth	100 (54 yrs)	RDBPCT	Patients with *H. pylori* infection	*L. reuteri* DSM17938	Not reported	37.5 mg probiotic tablet/b.i.d/1‐2wk**s**	Placebo**/**b.i.d**/**1‐2 wks	PPI‐ bismuth‐containing quadruple therapy with probiotic could provide an excellent cure for *H. Pylori* infection
						*L. reuteri* ATCC (PTA6475)				
Rosen et al. (2019) [36]	USA	Iron	52 (10.2 ± 4.2 yrs)	RDBPCT	Children with Iron deficiency	*L. plantarum* 299 v	10 × 10^9^ CFU	1 capsul**e/**d/	1 placebo**/**d**/**6‐8 wks	Probiotic LP299v did not enhance treatment
								6‐8 wks		
Skrypnik et al. (2019) [23]	Poland	Fe, Cu, Zn	90 = 45–70 yrs (LD = 56.88 ± 6.41 HD = 56.00 ± 6.56)	RDBPCT	Iron metabolism in obese postmenopausal women	*B. bifidum* W23	Group LD = 2.5 × 10^9^ CFU	Probiotic/o.d/sachets/12 wks	Placebo	Multi‐strain probiotic supplementation may influence iron metabolism in obese postmenopausal female patients.
		Iron				*B. lactis* W51			(Maize starch and	
		Copper				*Lc. lactis* W19			Maltodextrins)/o. D/sachets/12 wks	
		Zinc				*B. lactis* W52	Group HD			
						*Lc. lactis* W58	1 × 10^10^ CFU			
						*L. acidophilus* W37				
						*L. brevis* W63				
						*L. casei* W56				
						*L. salivarius* W24				
Axling et al. (2020) [37]	Sweden	Iron	42 (22.3 ± 3.5)	RDBPCT	Iron‐depleted women	*L. plantarum* 299 v	1 × 10^10^ CFU	1 capsule/d/0‐12 wks	1 capsule (iron alone)/d/0‐12 wks	The use of probiotics plus iron causes a more substantial and rapid improvement in iron status
Axling et al. (2020) [37]	Sweden	Iron	228 (30.4 ± 4.3 yrs)	RDBPCT	Non‐anemic, pregnant women.	*L. plantarum 299 v*	1 × 10^10^ CFU	1 capsule/ B.i.d/gestational week 10–12 until end of pregnancy or until	Placebo capsules (maize starch and magnesium stearate(b.i.d))	Intake of *L. plantarum 299 v* attenuated the loss of iron stores and improved iron status in healthy pregnant women
Márquez et al. (2021) [38]	Spain	Bismuth	*N* = 80 Median age in years (IQR) 50.50 (17.0)	RDBPCT	Patients with *H. pylori* infection	*L. reuteri*	Not reproted	Three capsules four times a day, plus omeprazole 40 mg twice a day for ten days.	Maltodextrin in the control arm for 30 days	Treatment with *L. reuteri only* reduced abdominal pain and distension.
									Placebo.	
Sandroni A et al. (2021) [39]	USA		19	RDBPCT	Female athletes with iron supplementation	*B. lactis*	8 × 10^9^ CFU	(5 g prebiotic fiber	Placebo/ 8 wks	Synbiotic supplementation along with feso4 improved athletes' Fe status over 8 weeks
		Iron						140 mg ferrous sulfate, feso4/d)/8 wks		
Orlandoni et al. (2021) [40]	Switzerland	Zinc Selenium	32(mean age 79.7 ± 10.3 years),	Pilot DBPCT	Elderly people with feeding tubes	*L. plantarum*	10^12^ CFU	Proxian/60 d	Placebo/60 d.	Affect the modulation of inflammation and reduce the incidence of infections,
						*L. buchneri*				
						*B. animalis* subsp lactis				
Feng et al. (2022) [41]	China	Copper Nickel	152	RDBPCT	Workers from the metal industry	*L. bulgaricus S. thermophilus P. acidilactici GR‐1*	1 × 10^10^ CFU	12 wks/250 g/yogurt Daily	Conventional yogurt containing *L. bulgaricus* *S. thermophilus*	The use of probiotic yogurt may be an effective and affordable approach for combating toxic metal exposure through the protection of indigenous GM in humans.
Giorgetti et al. (2022) [42]	Switzerland	Iron	30 (26.2 y)	RSBCT	Iron‐depleted women	GOS and FOS	15 g GOS 15 g FOS	Days 1 and 22, 57fefum	Sucrose and lactose 6.1 g sucrose 1.5 g lactose/43 d	GOS and FOS may be promising new enhancers of supplemental iron absorption
								Days 4 and 25, 58fefum		
								15 g GOS 15 g FOS 15 g acacia gum/43 d		
He et al. (2022) [43]	China	Bismuth‐containing quadruple therapy	Probiotic group (*n* = 140) placebo group (*n* = 136)	RDBPCT	Patients with *H. pylori* infection	*B. tetragenous*	Not reported	14‐day	Placebo 28 days	The incidence of gastrointestinal adverse events was lower in probiotics group compared to placebo group
Bismuth‐containing quadruple therapy (esomeprazole, bismuth, amoxicillin, furazolidone)										
He et al. (2022) [44]	China	Bismuth quadruple therapy	168	Not reported	Patients with *H. pylori* infection	*S. boulardi*	500 mg bid of S. Boulardii powder	40 mg bid of pantoprazole sodium enteric tablets 2 weeks	Bismuth quadruple therapy	Bismuth quadruple therapy can lead to intestinal flora disorders in caga + /vaca s1m1 *H. pylori* patients. *S. boulardii* can improve the distribution
Viazis et al. (2022) [45]	Greece	Non‐bismuth quadruple	741	RBCT	Patients with *H. pylori* infection	*L. aidophilus*	1,75 × 10^9^CFU	Probiotic/b.i.d/15 d	Placebo/b.i.d/15 d	Probiotics to the 10‐day concomitant non‐bismuth quadruple *H. pylori* eradication regimen increases the eradication rate and decreases side effects.
						*L. plantarum*	0.5 × 10^9^ CFU			
						*B. lactis*	1,75 × 10^9^ CFU			
						*S. boulardii*	1, 5 × 10^9^ CFU			
Hemphill et al. (2023) [46]	USA	Iron	Probiotic group= 12	RDBPCT	Pregnant women with iron deficiency anemia	*L. plantarum 299 v*	Not found	Probiotic LP299V + PNVI/ (15–20 wks)	Placebo +PNVI/ (15–20 wks)	LP299V® may be a tolerable therapy during pregnancy and has the potential to affect maternal and neonatal hematological and iron status
			Placebo group= 8 (28.9 ± 6.5 yrs)							
Abdulah. et al. (2024) [47]	Iraq	Zinc	100	RCT	Children with mild or moderate to severe acute gastroenteritis	*B. infantis* *L. paracasei* *L. rhamnosus*	10 × 10^6^ CFU	Units/day1 wk/	Placebo/1 wk	Probiotics plus zinc did not significantly affect disease severity in children with gastroenteritis at 2 weeks.

*Note:* b.i.d: twice daily; d: day; FeSO_4_: ferrous sulfate; FOS: fructooligosaccharide; GOS: galactooligosaccharide; h: hours; LC: low calcium; M: months; o.d: once daily; ORS: oral rehydration solution; PNVI: prenatal vitamin with iron; PSCS: prospective single‐centre study; PW: pregnant women; RC: regular calcium; RCT: randomized controlled trial; RDBPCT: randomized, double‐blind placebo‐controlled trial; ROLPS: a randomized open‐label pilot study; RSBPCT: randomized single‐blinded placebo‐controlled trial; RSBCT: randomized, single‐blind, controlled trial; RTBPCT: randomized triple‐blind placebo‐controlled trial; SAC: school‐aged children; SBRPT: single‐blinded randomized prospective trial; SCRP; single‐centre, randomized, parallel; wks: weeks; XOS: xylooligosaccharides; yrs: years.

A total of 22 different probiotic species were administered once, twice, or three times daily at 1 × 10^7^ to 2.5 × 10^10^ colony forming units (CFU)/daily and an optimum dose of 3.13 × 10^9^ CFU/daily. *L. plantarum* and *L. acidophilus* were the most common probiotic species used in different studies reviewed. As shown in Table 1, 14 of the 31 clinical trials using probiotic strains used only a single bacterial strain. Also, eleven trials used a combination of multi‐strain probiotics.

### 3.1 Effectiveness of Probiotics or Prebiotics in Combination with Zinc

A total of eight clinical trials used probiotics or prebiotics in combination with zinc for the treatment of diarrhea [7, 8, 48, 49, 50, 51, 52, 53]. One trial [54] evaluated the immunogenicity of the oral rotavirus vaccine. Two [48, 50] of the six clinical trials evaluating the effect of probiotics plus zinc in the treatment of diarrhea showed a significant reduction in time to resolution of vomiting in the group receiving probiotics plus zinc compared with the group receiving zinc alone (0.35 h vs. 0.74 h; *p* < 0.05). Also, the time to resolution of diarrhea was significantly reduced in the probiotics plus zinc group compared to the zinc only group (2.22 days vs. 3.66 days) (*p* < 0.05). However, Dalgic et al. [48] indicated that there was no statistically significant difference (*p* > 0.05) between the probiotics plus zinc group and the zinc‐only group in time to resolution of diarrhea (3.11 vs. 3.41 days) and duration of hospitalization (4.11 vs. 4.33 days). Two clinical trials demonstrated the efficacy of the combination of probiotics and zinc compared to probiotics alone in the treatment of diarrhea [7, 8]. In addition, Abdulah et al., [8] showed that the incidence of dehydration was lower in the probiotic plus zinc group than in the probiotic group (90% vs. 76.47%), while there was a greater reduction in the incidence of gastroenteritis in the probiotic group than in the probiotic plus zinc group (90.2% vs. 76%). Children in the probiotics plus zinc group had a significantly shorter time to resolution than those in the probiotics group, 1.34 versus 2.00 days (*p* < 0.001). On the other hand, Xiang et al., [7] reported the beneficial effects of probiotics plus zinc on antibiotic‐associated diarrhea secondary to pneumonia in children. They indicated that the treatment efficacy rate was significantly higher in the probiotics plus zinc group than in the probiotic group (92% vs 68% *p* = 0.02). In addition, the mean length of hospital stay was lower in the probiotics plus zinc group than in the probiotics group (6.12 days vs. 5.44 days *p* > 0.05), but this was not significant.

On the other hand, three [48, 49, 50] of the six clinical trials evaluated the effect of ORS fortified with zinc and probiotics/prebiotics on the treatment of diarrhea, two of which compared the effect of ORS fortified with zinc and probiotics/prebiotics with the effect of ORS alone (control group). The percentage of resolution of diarrhea during 72 h in the group receiving ORS enriched with zinc and probiotics was 72.15% compared to 53.25% in the group receiving ORS alone, indicating the significant effect (*p* < 0.05) of enriched ORS on the treatment of diarrhea. The number of watery or soft stools was also significantly (*p* < 0.05) reduced in the group receiving ORS fortified with zinc and probiotics compared to the group receiving ORS alone (1.67 vs. 2.29). Parents of children receiving fortified ORS also missed fewer days of work than those receiving ORS (1.09 vs. 1.42; *p* > 0.05). However, Maragkoudaki et al. found that the number of work days missed for infant care was lower in the fortified ORS group than in the ORS group (1.8 vs. 3 days); but this difference was not statistically significant (*p* > 0.05) [49]. Passariello et al. [48] reported a greater need for additional supportive care 72 h after the intervention in patients receiving ORS compared to those receiving enriched ORS (31.6% vs. 10.1%, *p* = 0.004).

On the other hand, Yazar et al. [48] compared the effect of a synbiotic preparation and a zinc suspension separately on the duration of diarrhea; the results indicated that the duration of diarrhea was significantly (*p* < 0.001) reduced in both the synbiotic (91 h) and zinc‐enriched ORS (86 h) groups compared to the ORS control group (114 h). Also, the percentage of children with diarrhea in the zinc‐fortified ORS group was lower than in the synbiotic group 72 (45.4% vs. 61.8%, *p* < 0.05) and 96 (14.5% vs. 27.2%, *p* < 0.05) hours after the intervention, indicating that zinc‐fortified ORS was more effective than synbiotic. Sheele et al. [49] also evaluated the effect of bismuth subsalicylate and probiotics on cholera patients and found that neither bismuth subsalicylate nor probiotics or bismuth subsalicylate plus probiotics was effective in reducing the duration and severity of cholera. Lazarus et al. [50] showed that probiotic supplementation plus zinc improved the immunogenicity of the oral rotavirus vaccine by 43.8% compared with the placebo group. However, zinc or probiotic supplementation alone had no significant effect on rotavirus vaccine immunogenicity (*p* > 0.05).

### 3.2 Effect of Probiotics or Prebiotics on Iron Status

**Figure 2:**
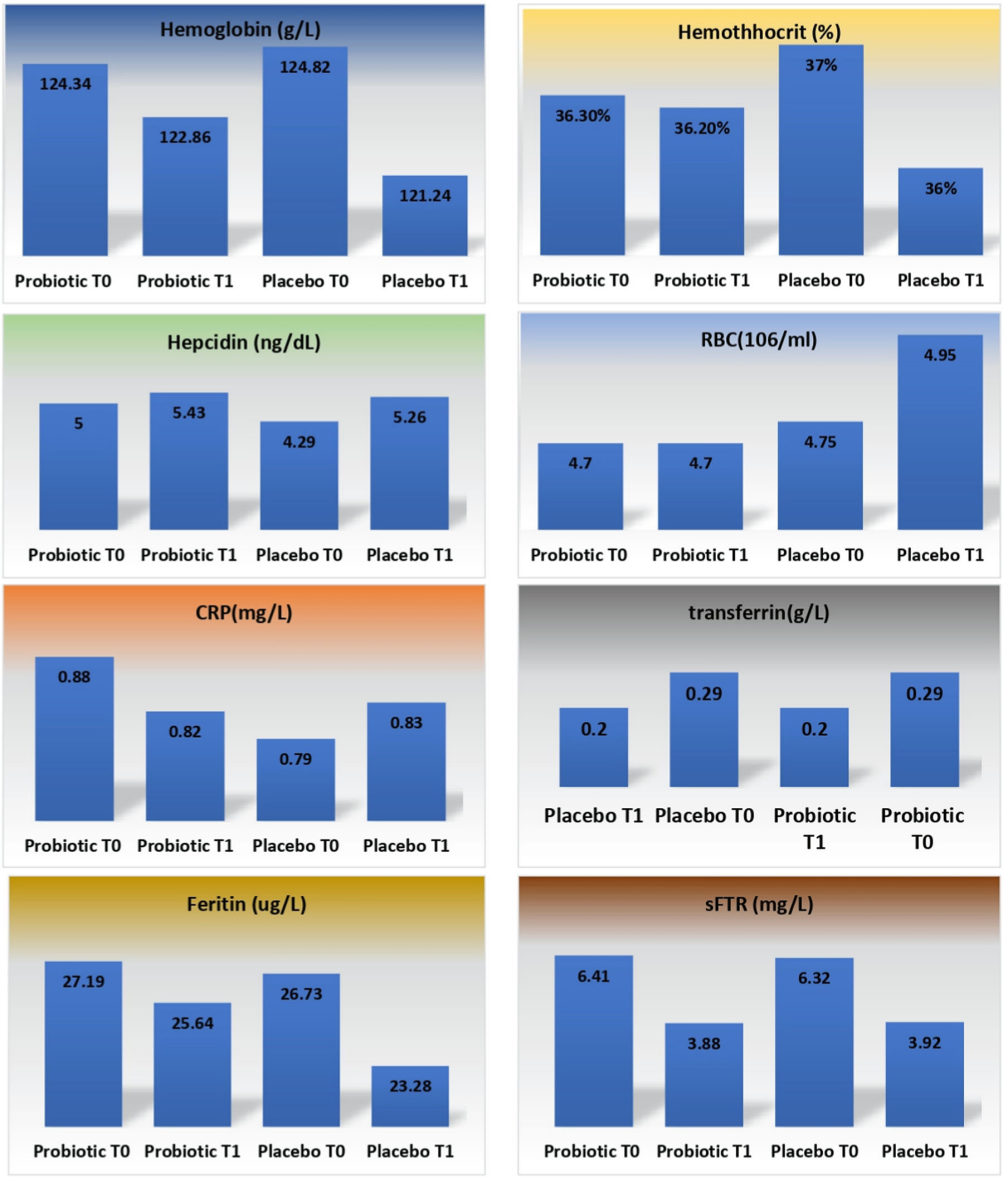
Comparing some morphological and biochemical parameters before (T0) and after (T1) the intervention between the placebo and probiotic groups. a: CRP, b: hemoglobin, c: Hepcidin, d: Feritin, e: Transferrin, f: sFTR, g:RBC, h: Hematocrit.

The effects of probiotics or prebiotics on iron status in blood samples were evaluated in eleven clinical trials [48, 49, 50, 51, 52, 53, 54, 55, 56, 57, 58], of which seven trials focused on iron metabolism, two trials focused on fractional iron absorption (FIA) and two trial focused on iron bioavailability [57, 58]. On the other hand, prebiotic was used in four out of these ten trials [52, 55, 56, 57]. The probiotic strain used in four clinical trials was *L. plantarum* 299 v [48, 49, 51, 53]. As shown in Figure 2. some morphological and biochemical parameters were compared before (T0) and after (T1). The probiotic group's serum hepcidin concentration increased by 8.6% after the intervention, whereas the placebo group's increased by 22.61%. Also, plasma ferritin concentrations decreased in both probiotic and placebo groups after the intervention (5.7% vs. 12.9%); and it was statistically significant (*p* < 0.05). Also, sFTR concentrations decreased in both probiotic and placebo groups after the intervention (39.4% vs. 37.9%%); and it not was statistically significant (*p* < 0.05). Furthermore, the mean percentage of RBCs (red blood cells) in the placebo group did not change after the intervention, whereas it increased by 4.21% in the probiotic group. The mean percentage of hemoglobin, hematocrit, and CRP did not change after the intervention in any of the probiotic and placebo groups.

The probiotics had a dual effect on bioavailability of nonheme Fe sources and heme‐Fe. Moreover, Sandroni et al., [58] indicated that synbiotic supplementation along with FeSO4 significantly improved serum ferritin levels in the supplementation group after 8 weeks compared to placebo (122% vs. 209%, *p* < 0.05), indicating the enhancement of bioavailability of nonheme Fe sources in the combination with probiotics. While, Weinborn et al., [57] indicated that the consumption of prebiotics increased heme Fe bioavailability by 65% after the intervention, whereas it did not affect nonheme Fe bioavailability. Giorgetti et al., [56] evaluated that the effect of prebiotic galacto‐oligosaccharides (GOS) on the increase of iron absorption from ferrous fumarate (FeFum) given to women and children during 14 days. They indicated that fractional iron absorption from FeFum given with GOS and FOS was significantly higher ( + 33.3% and +34.8%, respectively; *p* < 0.001 for both) than control (23%).

### 3.3 Effects of Probiotics on Bismuth‐Containing Quadruple Therapy for *Helicobacter pylori* Infection

**Figure 3:**
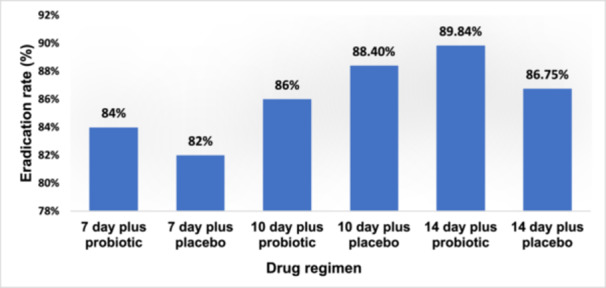
Eradication rates of *H. pylori* infection in regimes containing probiotics with and without bismuth‐containing quadruple therapy.

The effects of probiotics on bismuth‐containing quadruple therapy for *H. pylori* infection were evaluated in seven clinical trials [59, 60, 61, 62, 63, 64, 65]. Figure 3 compares the eradication rates of *H. pylori* infection in the bismuth‐containing quadruple therapy regimen with and without probiotics. According to these results, there was no significant difference in the mean eradication rate of *H. pylori* infection (*p* > 0.05) between the 7‐day, 10‐day and 14‐day regimens containing probiotics and a regimen without probiotics. Table 2 compares the adverse events of the 7‐day and 14‐day probiotic and non‐probiotic regimens. According to these results, some complications of the bismuth‐containing quadruple therapy regimen, such as bitter taste, diarrhea, vomiting, nausea and abdominal pain, were less frequent in the probiotic group than in the placebo group.

**Table 2:** Frequency of adverse events of probiotic and non‐probiotic regimen.

	A 7‐day regimen plus probiotic	A 7‐day regimen plus placebo	A 10 or 14‐day regimen plus probiotic	A 10 or 14‐day regimen plus placebo
Bitter taste	27%	38%	16.3%	**30.79%**
Diarrhea	12%	14.5%	10.97%	**15.87%**
Vomiting	18%	20%	7.19%	**12.51%**
Nausea	18%	20%	8.96%	**19%**
Abdominal pain	4%	10%	10.13%	**14.03%**

### 3.4 Effect of Probiotic Supplementation on Patients With Systemic Nickel Allergy Syndrome

Randazzo et al., [66] showed an increase in the diversity of lactic acid bacterial populations in fecal samples from patients receiving probiotic supplements. Gastrointestinal reactions also improved significantly (*p* < 0.05) only in the probiotic group. However, the severity and frequency of cutaneous symptoms such as urticaria, itching, and eczema were statistically reduced (*p* < 0.05) in both the probiotic and placebo groups.

### 3.5 Influence of Probiotics on Toxic Metal Levels

According to Bisanz et al. [67], probiotics significantly reduced levels of toxic metals such as lead, mercury, and arsenic in pregnant women and school children. In the control group of pregnant women, mercury and arsenic levels increased by 34% and 10.4%, respectively (*p* > 0.05). These concentrations remained stable in the probiotic group, indicating a protective effect of probiotic consumption. They also showed no significant difference in blood levels of arsenic, mercury, lead and cadmium (*p* > 0.05) between schoolchildren receiving probiotics and the control group. However, we observed a weak decreasing trend in blood levels of arsenic in the probiotic group. Feng et al., [67] indicated that the consumption of probiotic yogurt in 152 occupational workers and 30 adult students after 12 weeks group caused a reduction of blood Cu and Ni levels by 34.45% (from 1246 ± 207.7 to 817.0 ± 145.1 μg/L) and 38.34% (from 4,855 ± 3,392 to 2,994 ± 1,942 μg/L), respectively, which were more significant than those noted in the conventional yogurt group. Conversely, the Cu and Ni levels in fecal samples of subjects with consumption of probiotic yogurt increased compared to those with consumption of conventional yogurt (*p* < 0.001) after 12 weeks of intervention, indicating increased excretion of these heavy metals with probiotic yogurt ingestion.

### 3.6 Effect of Probiotics in Reducing the Incidence of Infection and Modulating Inflammation in Elderly Patients With Feeding Tubes

Orlandoni et al., [40] found that the incidence of bacterial infections was lower in the probiotic plus zinc gluconate (1.5 mg) and sodium selenite (27.5 mcg) group than in the placebo group, but the observed difference was not statistically significant (25% vs. 44%, *p* = 0.229). Similarly, antibiotic therapy (12% in the probiotic group vs. 37% in the placebo group, *p* = 0.110) and modulation of CRP levels (median CRP moved from 0.95 mg/L (t0) to 0.7 (t1) in the probiotic plus zinc group versus 0.7 mg/L in the control group) did not reach statistical significance.

## 4 Discussion

Xenobiotics are generally not found in living organisms, and their presence could cause inflammation, endocrine disruption, oxidative stress, cancer, and intestinal problems. Heavy metals are a type of xenobiotic [68]. Due to heavy metals' importance as essential and toxic elements, this study examined how probiotics affect heavy metals in humans.

Consumption of water or food contaminated with heavy metals by humans and their entry into the stomach's acidic environment causes the metals to become acidic and turn into oxidative forms. The resulting radicals can bind tightly and stably to various molecules in bio‐systems such as proteins and enzymes [4, 69]. Heavy metals are attached to functional thiol groups (‐SH group of cysteine and –SCH 3 group of methionine) [4]. Some heavy metals, such as iron, chromium, cobalt, and arsenic produce free radicals that cause oxidative damage to DNA and cells. Oxidation of heavy metals often induces the formation of free hydroxyl radicals. The free radical attacks the lipid membrane and forms a lipid radical that reacts with dioxygen or lipid. This reaction causes damage to the fat molecule. Eventually, two lipid radicals react with lipid peroxyl radicals to form a stable lipid [4]. The intestinal microflora is the primary target of heavy metals, which disturb the composition and synthesis of metabolites [70]. Heavy metals can lead to neurotoxicity by reducing neurotransmitters or accumulating in the mitochondria of neurons, disrupting DNA synthesis [4]. In addition, heavy metals alter the intestinal microbiota's composition and metabolic profile [71]. Probiotics could affect heavy metals due to some of their unique properties as follows: (1) the ability to bind, tolerate, or detoxify heavy metals, (2) high tolerance to acid and bile, which causes probiotics to work well in the gastrointestinal tract, (3) the ability to bind to the intestine [4], (4) acting as potent antioxidants (butyrate, folate, vitamins B1 and B12, or oxidative enzymes) [72], and (5) inducing regulatory immune responses [4, 73]. Probiotics exhibit antioxidant effects by inhibiting lipid peroxidation activity, scavenging free radicals, and reducing DNA legions in host tissues [68]. One of the world's environmental problems today is heavy metal pollution. Heavy metals cause danger to humans through the food chain and cause a series of serious diseases in humans [67]. Heavy metal detoxification by probiotics is done through several mechanisms as follows: (1) attachment of metal ions to the bacterial cell wall, their passage through the cell membrane, entry into the cell, and accumulation inside the cell, this process is called bioaccumulation [74]; (2) conversion of more toxic forms of heavy metals to less toxic forms, as lactobacilli could convert methylmercury to mineral mercury and then to Hg0, which is less absorbed in the gastrointestinal tract [75]; (3) modulation of intestinal microflora composition; (4) reduction of oxidative stress [68]; (5) increased function of intestinal barriers and regulation of tight junction of small intestinal epithelium that restricts heavy metals entry into the systemic circulation [68, 69]; (6) modulation of host gene expression; (7) effect on host abilities to metabolize xenobiotics [68]; (8) competition with heavy metals for intestinal absorption [73]; (9) stimulation of intestinal peristalsis, which causes the excretion of xenobiotics through the stool [76]; and (10) binding (lactobacilli) to cationic heavy metals. Binding depends on the type of microbial strain and pH. Binding mainly occurs at pH 4‐6 [77]. Ameen et al. showed that *L. plantarum* attached better to heavy metals in acidic environments [78]. Heat is another critical factor in binding heavy metals to the surface of bacteria. Increasing the temperature increases the removal of cadmium [77]. Also, increasing the bacterial concentration increases the binding of cadmium and lead. Heavy metals are absorbed into organic acids present in bacterial surface structures (e.g., polysaccharide capsules and teichoic acids in *L. fermentum* ME3 and *Bifidobacterium longum* 46 [79] and reduce available bond levels [77]. If chemical changes neutralize the negative charge of the carboxyl and phosphoryl groups, the binding of heavy metals to *L. fermentum* ME3 and *B. longum* 46 is reduced because cationic metals tend to bind to negative charges. Both carboxyl and phosphoryl groups are important binding sites and may be involved in ion exchange [77]. Lead binding to *L. fermentum* ME3 and *B. longum* 46 surfaces has been confirmed [77, 79].

Cadmium and lead levels in water should not exceed 3–10 g/L [77]. Exposure to cadmium causes diseases, including kidney damage, osteoporosis, increased bone excretion, and lung cancer [77]. One of the effects of cadmium is the inhibition of thiol transferase [4]. A study showed that genetically modified *Escherichia coli* isolates reduced rats' cadmium‐ and mercury‐induced liver and kidney damage [80]. Arsenic is a metalloid found in water, soil, rocks, and living organisms. Side effects of arsenic include skin lesions and cancers of the lungs, bladder, and kidneys [77]. Adding amino acids to L. casei accelerates the removal of arsenic, indicating that arsenic binds to the surface of the bacteria [77]. *Faecalibacterium prausnitzii* produces methyltransferase that detoxifies arsenic [4]. Only one clinical trial, among the studies reviewed in this research examined the inhibitory effects of probiotics on toxic heavy metals; this clinical trial found that *L. rhamnosus* GR‐1 reduced arsenic and mercury levels in pregnant women but not in children. Due to the scarcity of such studies, it is suggested that more extensive clinical trials be conducted to evaluate the efficacy of probiotics in inhibiting and reducing toxic heavy metals in the blood in different age groups and communities.

Zinc is one of the essential elements needed for the normal functioning of several enzymes. It is less toxic than other heavy metals; however, overuse could cause kidney failure and damage the pulmonary alveoli [81]. One of the most common problems of children in developing countries is zinc deficiency. However, the protective effect of zinc is not fully understood. The role of zinc in human cell growth, differentiation, DNA synthesis, improving water and electrolyte absorption, and enhancing immune function has been identified [82]. *L. fermentum* and *L. plantarum* actively remove zinc in an aqueous solution [83].

Zinc is currently on the World Health Organization (WHO) list of essential medications for diarrhea. Also, in the Copenhagen Consensus in 2008, zinc supplementation was considered as the most effective intervention in promoting human development [84]. In infants and children with acute diarrhea, zinc supplementation reduces the severity and duration of diarrhea [85]. Many studies have been conducted on the effectiveness of zinc in treating acute diarrhea in children, but the results are inconsistent according to geographical area and age [86]. Immune status and weight are two essential factors in determining the duration of diarrhea. Zinc is vital in determining people's immune status and weight in developing countries [86]. The results of one study showed that oral zinc supplementation reduced the output and frequency of stool in patients with acute diarrhea [87].

Nevertheless, the results of a meta‐analysis showed that zinc consumption reduced the duration of diarrhea, but had no effect on the output and frequency of stool [88]. Another study showed that the combination of zinc and probiotics reduced the severity and duration of diarrhea [89]. In a study evaluating the prophylactic effect of zinc and probiotics on rhesus monkeys, it was found that taking probiotics alone reduced the severity of diarrhea, but adding zinc to probiotics had no additional beneficial effects [90]. A meta‐analysis examining the effectiveness of various interventions in treating pediatric diarrhea showed that the best intervention was using a combination of *Saccharomyces boulardii* and zinc [91]. In the present study, contradictory results were observed regarding reducing the duration of diarrhea. While some studies showed the effectiveness of probiotics and zinc in reducing the duration of diarrhea, others showed no beneficial effect. This may be due to differences in the type of probiotics, probiotic dose, and zinc dose used in different studies. ORS and zinc supplementation apply to malnourished children with acute diarrhea [92]. In all the studies reviewed in this research, using a combination of probiotics, zinc, and ORS reduced the disease severity.

Drinking water can contain no more than 0.3 mg/L [47] of iron, according to the WHO. Iron is necessary for oxygen transport and storage, hormone synthesis, DNA replication, cell cycle regeneration and control, and nitrogen fixation in the body [93]. Cancer, kidney problems, and metabolic acidosis are caused by iron accumulation in the body. *L. fermentum* is involved in iron absorption [81]. It has been found that 38% of pregnant women in reproductive age suffer from anemia. Various causes have been reported for anemia, such as iron deficiency, inherited blood diseases, malaria, exposure to hazardous chemicals, and schistosomiasis [94]. Iron deficiency accounts for 50% of all anemia cases among the various factors. Persistent iron deficiency could lead to serious health problems. There are several ways to treat iron deficiency, such as proper nutrition, intramuscular injections, supplements, and iron‐rich diets [95]. Ferritin levels are considered a marker of iron deficiency [96]. There is a synergy between probiotics and prebiotics, which increases the amount of heme iron in the body [94]. According to the information obtained from a systematic review and meta‐analysis, the probiotic Lp299v was found to affect iron absorption from diets significantly. Various mechanisms have been proposed for the effect of Lp299v on iron absorption, including (1) the production of p‐hydroxyphenyl acetic acid as a microbial product that reduces the ferric form of iron into the bioavailable ferrous form, (2) increasing mucin production at the intestinal surface, which induces iron uptake by enterocytes, and (3) the production an anti‐inflammatory, immune response that induces the suppression of hepcidin [9]. Hepcidin is the primary regulator of systemic iron homeostasis, which promotes iron bioavailability [97]. Hepcidin regulates the expression of FPN1 (iron exporter), which causes iron to enter the bloodstream [96].

Iron is essential in all biological systems. It is important in oxygen transport, DNA synthesis and cellular respiration [53]. Various mechanisms have been proposed for the action of prebiotics in iron absorption. First, prebiotics increases iron absorption by producing osmotically active sugars produced during the fermentation of prebiotics. These sugars increase the inactive absorption of iron and produce weak organic acids that facilitate the absorption of minerals [98]. Second, the production of organic acids lowers the pH, which facilitates the conversion of Fe^3 +^ to Fe^2 +^ [99]. Third, prebiotics are fermented by the colon microbiota and produce short‐chain fatty acids, such as acetate, propionate, and butyrate, which increase the proliferation of epithelial cells and ultimately increase absorption, especially iron absorption [100]. Fourth, if prebiotics are taken with iron, the expression of the HAMP gene involved in regulating iron absorption is increased. Fifth, the concentration of circulating hepcidin decreases due to the anti‐inflammatory effects on the colon [101]. Inulin and GOS (Galactooligosaccharides) are listed by the Food and Drug Administration (FDA) of the United States as GRAS (generally recognized as safe), and both increase iron absorption [102]. Inulin is one prebiotic used in the clinical trials reviewed in the present study. The results of an in vitro study investigating the effect of inulin on iron availability in yoghurt showed that inulin alone was not effective in improving iron absorption but exhibited beneficial effects when used with probiotics [103]. The present study found that consuming a diet containing inulin improves intestinal absorption of iron by reducing hepcidin but has no effect on other factors such as ferritin, hemoglobin, and CRP. Inulin has also been shown to be effective in improving heme Fe bioavailability. Due to the lack of sufficient studies, it is suggested that more extensive clinical trials be conducted to evaluate the effect of inulin and other prebiotics on ferritin levels and other factors involved in iron absorption.

Bismuth has long been known to have anti‐*H. pylori* properties. No resistance to bismuth has been reported in *H. pylori* isolates [104]. Administration of bismuth in quadruple therapy improves the eradication rate of *H. pylori* by up to 90% [105]. Adding probiotics to conventional therapies contributes to better eradicating *H. pylori* infections [106]. The present study results revealed that among the three clinical trials evaluating the effectiveness of probiotics with quadruple therapy, only two studies reported the beneficial effects of probiotics. No beneficial effect was observed in a study conducted in Iran [61]. Differences in these studies' results may be due to differences in probiotics, probiotic dosage, and individual differences in target communities, such as genetic and nutritional differences. However, probiotics have been shown to improve the eradication rate of *H. pylori* and reduce side effects when consumed with a quadruple diet [107].

In the studies reviewed, the probiotics' dose ranged from 10^8^ to 10^10^, and various bacteria and even yeast were used as probiotics. Bacteria that affect heavy metals include *L. reuteri*, *L. plantarum*, *L. acidophilus*, *B. lactis*, *L. brevis*, *L. casei*, *L. paracasei*, *L. salivarius*, *L. rhamnosus*, *B. bifidum*, *B. breve*, *L. lactis, L. bulgaricus, B. longum, S. boulardii* and *S. thermophilus*. Although these probiotics showed beneficial effects in some of the reviewed studies, no beneficial effects were reported in others, especially in treating cholera and *H. pylori* infections and iron deficiency. Therefore, there is a need for more extensive studies in this field.

The limitations of the present study are as follows. First, among the reviewed studies, some heavy metals were less studied or not studied, indicating the need for further studies. Second, studies were limited to some parts of the world. Given that the type of nutrition affects microbiota composition, it is better to do more extensive studies in different communities and regions worldwide. Third, in some areas, such as the effect of probiotics on lowering blood levels of heavy metals, the clinical trials were so few that it was impossible to conduct a detailed study and meta‐analysis. Fourth, some aspects of iron status and absorption were overlooked, and further studies are needed, such as investigating the impact of social status and nutrition and the long‐term effects of probiotics.

## 5 Conclusions

Today, humans are exposed to various environmental and chemical pollutants produced through various industries and agriculture. Heavy metals are one of the most important contaminants in food. Contamination by these substances could have economic and public health consequences in different countries. Today, probiotics are one of the new methods employed to improve or eliminate the adverse effects of heavy metals in the body. Probiotics could induce their positive effects through a variety of mechanisms. The positive effects of probiotics and prebiotics in treating or reducing the severity of diarrhea in patients, beneficial effects in iron absorption, improving *H. pylori* treatment methods, positive effects on improving the symptoms of nickel allergy syndrome and reducing the levels of heavy metals in pregnant women, are cases that have been proven. Although many studies have investigated the effects of probiotics on heavy metals, there is still a need for more in‐depth and extensive studies.

## Author Contributions

**Atieh Darbandi:** conceptualization, investigation, methodology, data curation, software, writing – original draft. **Tahereh Navidifar:** conceptualization, methodology, investigation, software, writing – original draft. **Maryam Koupaei:** conceptualization, methodology, investigation, writing – original draft. **Roghayeh Afifirad:** software, methodology, data curation. **Reyhaneh Amin Nezhad:** formal analysis, software. **Amir Emamie:** investigation, formal analysis, software. **Malihe Talebi:** conceptualization, methodology, validation, visualization, writing – original draft, writing – review and editing, project administration, resources, supervision. **Maryam Kakanj:** methodology, conceptualization, investigation, writing – original draft, writing – review and editing, project administration, software, data curation, supervision, validation.

## Conflicts of Interest

The authors declare no conflicts of interest and the funding source have not any role in study design; collection, analysis, and interpretation of data; writing of the report; and the decision to submit the report for publication.

### Transparency Statement

The Malihe Talebi affirms that this manuscript is an honest, accurate, and transparent account of the study being reported; that no important aspects of the study have been omitted; and that any discrepancies from the study as planned (and, if relevant, registered) have been explained.

## Data Availability

The data that support the findings of this study are available from the corresponding author upon reasonable request. The datasets generated and/or analysed during the current study are available from the corresponding author on reasonable request. by email. All authors have read and approved the final version of the manuscript. Corresponding author had full access to all of the data in this study and takes complete responsibility for the integrity of the data and the accuracy of the data analysis.
